# BNP as a potential biomarker for cardiac damage of breast cancer after radiotherapy: a meta-analysis

**DOI:** 10.1097/MD.0000000000016507

**Published:** 2019-07-19

**Authors:** Cheng Zhang, Dan Shi, Ping Yang

**Affiliations:** a Department of Cardiology, China-Japan Union Hospital of Jilin University; Jilin Provincial Key Laboratory for Genetic Diagnosis of Cardiovascular Disease; Jilin Provincial Engineering Laboratory for Endothelial Function and Genetic Diagnosis of Cardiovascular Disease; Jilin Provincial Molecular Biology Research Center for Precision Medicine of Major Cardiovascular Disease; Jilin Provincial Cardiovascular Research Institute; b Department of Radiation Oncology, China Japan Union hospital of Jilin University, Changchun, China.

**Keywords:** BNP, breast cancer, radiotherapy

## Abstract

**Background:**

To analyze whether BNP could be a potential biomarker for cardiac damage of breast cancer after radiotherapy.

**Methods:**

PubMed, Web of Science, ProQuest and Medline were searched using the key words “breast cancer” (“breast tumor”, “breast neoplasm”, or “breast carcinoma”), “brain natriuretic peptide” (or BNP) and “radiotherapy” (or “radiation therapy”). Four articles were selected and analyzed using the STATA 12.0 software package. The standard mean difference (SMD) and its standard error for BNP were calculated to assess the relationship between BNP and radiotherapy for breast cancer patients.

**Results:**

In total, 172 patients with breast cancer were identified. The pooled SMD was -0.233 (95% CI −1.113, −0.057). The pooled estimated SMD for all studies showed obvious significant difference (z = 3.99, *P* = .000). There was no publication bias.

**Conclusions:**

This meta-analysis suggested that BNP could be a biomarker of cardiac damage at high heart absorbed doses according to radiotherapy, especially for left breast cancer patients.

## 1 Introductions

Breast cancer is the most common cancer and the leading cause of cancer death among women worldwide. Chemotherapy and radiotherapy play crucial role in local control and metastasis of breast cancer. However, it has been reported that cardiovascular mortality are observed in breast cancer patients especially for left breast cancer after chemotherapy and/or radiotherapy. Cardiac biomarkers such as brain natriuretic peptide (BNP) and troponin (TnI) may be used to monitor cardiotoxicity and assess early signs of cardiovascular dysfunction. Plasma levels of TnI have been used as a prognostic marker of cardiac disease for high dose chemotherapy,^[1,2]^ especially for anthracyclines.^[1]^ However, 2 trials found that in left breast cancer patients, left ventricular ejection fraction (LVEF) did not significantly change after radiotherapy, and was not correlated with TnI levels.^[3,4]^ Hence, TnI is not considered for biomarker to cardiac damage of breast cancer patients.^[5–9]^ So, what about BNP? Could it be suitable? There had been already some articles about BNP as biomarker for heart damage in breast cancer patients. We conducted this meta-analysis to evaluate its potential role for biomarker, especially the patients who received radiotherapy and chemotherapy.

## 2 Methods

This meta-analysis was performed according to the Preferred Reporting Items for Systematic Reviews and Meta-Analyses (PRISMA) guidelines.^[10]^ An ethical approval was not necessary.

### 2.1 Search strategy

We identified studies in PubMed, Web of Science, ProQuest and Medline based on combinations of the following keywords

“breast cancer” (“breast tumor”, “breast neoplasm”, or “breast carcinoma”), “brain natriuretic peptide” (or BNP) and “radiotherapy” (or “radiation therapy”). The most recent article was updated on 2016. We also manually searched the references of related articles in this analysis.

### 2.2 Inclusion/exclusion criteria

Studies were considered eligible if they met the following inclusion criteria: The studies involved patients with left breast cancer, without metastasis and recurrence. The patients underwent radical/conserving surgery followed by radiotherapy and/or chemotherapy. The articles were written as full papers in English.

Studies were excluded for the following reasons: The publications were review articles, letters, case reports, expert opinions, or meeting records. Non-human research was performed. Patients had recurrent or metastatic disease. Patients were with right breast cancer or cardiac dysfunction. The publications were not written in English.

### 2.3 Data extraction

To avoid the repeated inclusion of the same data, the largest study with the longest follow-up time was included if there were several published studies involving the same patients at the same research center. We included one study if different patients were included in 2 studies at the same research center. Similarly, when there were multiple sets of data in one study, such as subsets of patients with different stage disease, we listed all data in separate sets. For data extraction, eligible articles were reviewed independently by 2 investigators. Discrepancies were resolved by discussion between the reviewers prior to data extraction. In cases of different opinions, a third reviewer was consulted to reach consensus.

Means and standard deviations analyses from publications were included in our analysis. Mean and standard deviation were calculated by median, maximum, and minimum.^[11]^

Additional data were carefully extracted from all the eligible publications using a standardized data collection form, including first author, publication year, tumor stage, chemotherapy regimen, surgery type and other important clinical characteristics.

### 2.4 Statistical methods

We calculated the available statistics with SMD with 95% confidence intervals (95% CI) calculated by either random-effects model or fixed-effects model to evaluate the correlation and Z test was utilized to evaluate the effect size^[12]^ according to means and standard deviations from every studies.

Forest plots were used to estimate BNP changes after radiotherapy and/or chemotherapy for left breast cancer patients.

Heterogeneity was defined as *P* *≤* .10 or I^2^ > 40%. When homogeneity was present (*P* > .10, I^2^≤40%),^[13]^ a fixed effect model was used for secondary analysis. Publication bias is a major concern for all meta-analyses. Funnel plots were generated to assess potential publication bias, and *P* > .05 indicated no potential publication bias.^[14]^ All statistical analyses were conducted using the STATA 12.0 software package.

## 3 Results

### 3.1 Characteristics of identified studies

**Figure 1:**
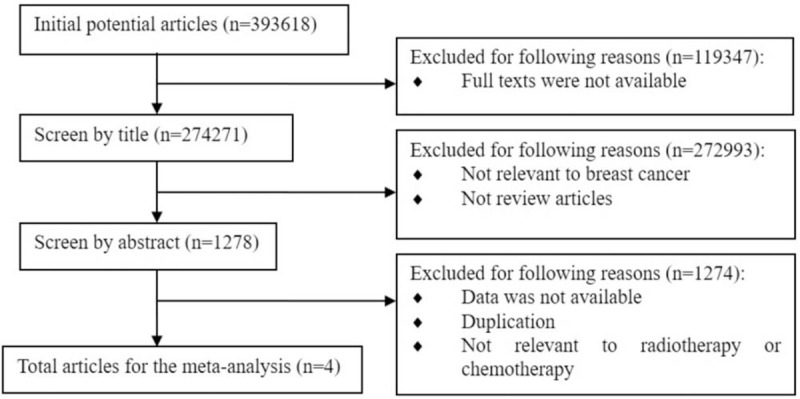
Flow chart of study identification.

According to our previously defined criteria, the initial electronic online search of PubMed databases, Web of Science, ProQest, and Medline retrieved 393,618 papers. After the selection according to the inclusive criteria, 4 eligible studies were finally included (Fig. 1).

There were totally 172 patients with breast cancer from the 4 included manuscripts. In Meinardi 2001, patients received radiotherapy after chemotherapy including left and/ or right modified radical mastectomy or breast-conserving treatment. In D’Errico 2012, patients with left breast conserving surgery with radiotherapy after chemotherapy were compared with no radiotherapy. In D’Errico 2015, patients with left breast conserving surgery or modified radical hysterectomy were concerned. The BNP data was collected according to different time after radiotherapy. In Palumbo 2016, patients with left breast conserving surgery were selected and BNP were evaluated according to different time after radiotherapy. According to radiotherapy, only 3-dimensional conformal technology in Palumbo 2016 was applied, other articles were not available (Table 1).

**Table 1:**
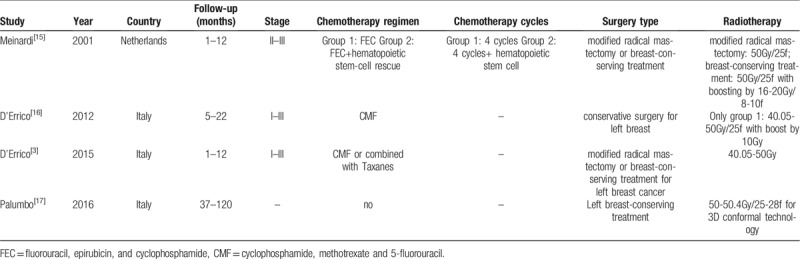
Main characteristics of the studies included in the meta-analysis.

### 3.2 BNP changes after radiotherapy/chemotherapy for breast cancer patients

**Figure 2:**
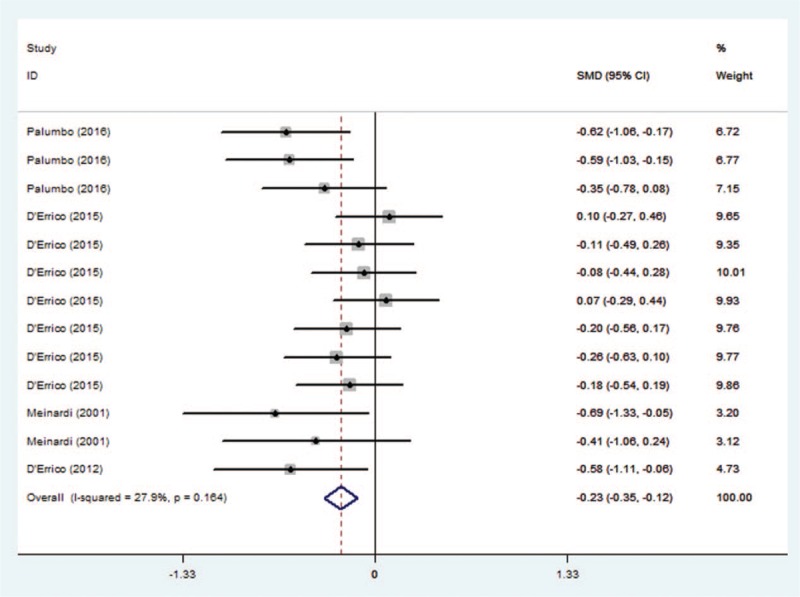
Results of the meta-analysis of BNP changes for breast cancer patients. SMD were available in 4 studies for a total of 172 patients. The pooled SMD was −0.233 (95% CI −1.113, −0.057). The heterogeneity among studies was not high (*I*^*2*^ = 27.9%, *P* = .164). The pooled estimated SMD for all studies showed obvious significant difference (z = 3.99, *P* = .000). BNP = brain natriuretic peptide, SMD = standard mean difference.

SMD were available in 4 studies for a total of 172 patients. The pooled SMD was −0.233 (95% CI −1.113, −0.057). The heterogeneity among studies was not high (*I*^*2*^ = 27.9%, *P* = .164). The pooled estimated SMD for all studies showed obvious significant difference (z = 3.99, *P* = .000) (Fig. 2).

### 3.3 Publication bias analysis

**Figure 3:**
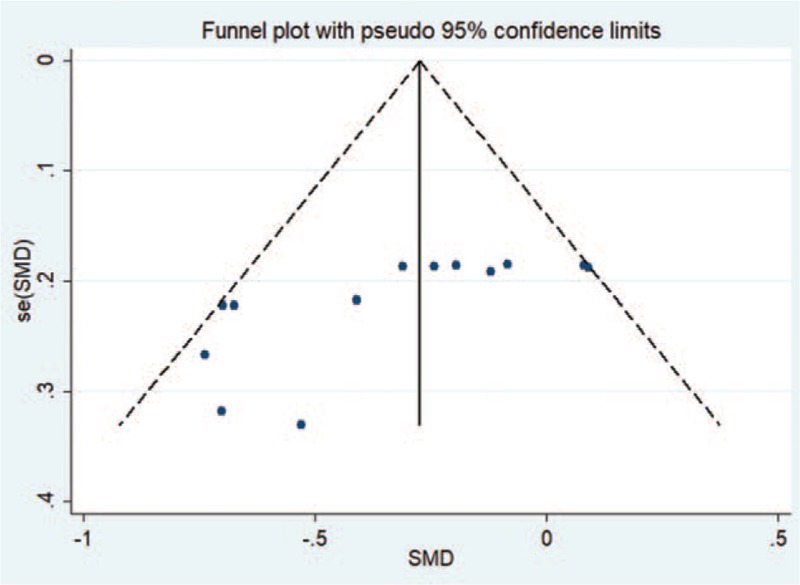
Funnel plots for publication bias. Funnel plots were generated to assess publication bias. These plots showed obvious symmetry and no publication bias in the studies.

Funnel plots were generated to assess the publication bias of the studies. These plots showed obvious symmetry and no publication bias (Fig. 3).

## 4 Discussions

BNP is an endogenous peptide produced initially by ventricular cardiomyocytes as a 134-aa pre-pro-peptide. Its levels were 20.3 (9.9–36.5) pg/ml and 21.1(9.8–37.7) g/ml in patients without any left ventricular (LV) dysfunction and 61.5 (50–68.4) pg/ml in patients with LV dysfunction^[5–9]^. BNP was secreted as a consequence of left ventricle impairment, and was elevated in heart failure and in acute coronary syndrome which includes acute myocardial infarction (MI) and unstable angina.^[5–9]^ BNP as a widely accepted marker of cardiac failure has widely used in clinical usage,^[5–9]^ especially in the diagnosis of heart failure.^[18,19]^ Cardiac dysfunction is one of complications which could affect patients survival quality, even modern radiotherapy improvements are applied.^[20]^ Biomarker for early diagnosis is emphasized to recognize the potential risk of cardiomyopathy, especially in left breast cancer patients.

According to this meta-analysis, breast cancer patients are involved, especially their treatments including surgery, chemotherapy, and radiotherapy. These treatments could induce the changes of BNP, which could be biomarker for breast cancer patients about cardiac failure. In this meta-analysis, most patients received conserving surgery for left breast according to these manuscripts. According to the results, the pooled SMD was −0.233 (95% CI −1.113, −0.057). The pooled estimated SMD showed obvious significant difference (z = 3.99, *P* = .000). These results showed that after radiotherapy, plasma levels of BNP was obvious increased especially in left breast cancer patients with significant difference. According to D’Errico 2015^[3]^ and Palumbo 2016,^[17]^ BNP levels were elevated after radiotherapy in 1 to 12 months and 37 to 120 months, respectively. These results reminded us that after radiotherapy, BNP variation arose earlier after treatment, and kept increasing in a very long time. However, the patients whose BNP elevated, could not be absolutely diagnosed as cardiac dysfunction. There is still no enough evidence to monitor the threshold of BNP by separating high risk potential patients. BNP could remind clinical recognition for further examinations and attentions. Hence, BNP could be a marker in follow-up for breast cancer after radiotherapy, as its stable and reliable characteristics. According to patients whose BNP elevated, more attention should be paid in case of cardiac dysfunction.

Radiotherapy plays a crucial role in early stage breast cancer after hysterectomy or conserving surgery with satisfying local control. Nevertheless, numerous epidemiological studies have shown that radiotherapy could increase cardiovascular mortality many years after treatment,^[21,22]^ especially for left- sided irradiation.^[23]^ The risk of cardiac mortality was 44% higher in left breast cancer patients than in those with right-sided in 6 to 24 months after radiotherapy.^[24–27]^ The early effects pericardial effusion, myocarditis and left ventricular dysfunction were induced after radiotherapy,^[28,29]^ is associated with the heart absorbed dose.^[29]^ Significant correlations between BNP and some dosimetric parameters of heart were found in those patients whose BNP values were above the threshold, and increasing correlation between BNP and hot spots of dose was showed.^[16]^ Higher values of D50% (Gy) seem to be related to BNP increase, even if it is not statistically significant. D50% (Gy) was the main parameter linked in a statistically significant. In addition, BNP values above the pathological cut-off threshold was correlated with high doses of radiation in small volumes (hot spots of dose), such as BNP and V_3Gy_%, D15 cm^3^ (Gy)/Dmean (Gy), D15 cm^3^ (Gy)/D50% (Gy) for the heart and V_2Gy_%, D1 cm^3^ (Gy)/Dmean (Gy), D 0.5 cm^3^ (Gy)/D50% (Gy) for the ventricle.^[3]^ Because 15 cm^3^ resulted in approximately 3% of mean heart volume, D15 cm^3^(Gy) indicates a dose near the maximum dose.^[30]^ When the ratios D15 cm^3^(Gy)/Dmean(Gy) and D15 cm^3^(Gy)/D50% (Gy) increased, BNP elevation indicated high dose radiation for heart. However, the methods for radiotherapy about 4 manuscripts were not available except Palumbo^[17]^ by 3-dimensional conformal radiotherapy using 2 tangential fields. Hence, intensity modulated radiation therapy (IMRT), proton beam irradiation, prone positioning and breath hold are recommended to reduce heart absorbed dose.^[31,32]^ In addition, partial breast irradiation as an alternative to whole irradiation after breast conserving surgery in selected patients,^[33]^ may reduce dose to the heart in those with left breast cancer.^[34]^ And their effects on BNP need further exploring. There is some relationship between BNP and radiation dose, which could induce heart injury. Hence, in order to control cardiac dysfunction, heart dose reduction is available, and BNP as a biomarker could monitor the effects of radiation on heart because of reflecting cardiomyocytes damage induced by radiation. Finally, the response ability of cardiomyocytes to radiation could be noticed and the possibility of heart failure could be foreseen according to the breast cancer patients.

European Society for Medical Oncology (ESMO) suggests measuring BNP to evaluate cardiotoxicity during and after radiotherapy for breast cancer patients.^[35]^ BNP measurement should be performed in patients with risk factors for cardiac damage, in those who receive high doses to the heart despite modern technology.^[36–39]^ According to follow up of breast cancer patients, BNP is a potential biomarker to heart damage, and is recommended to detect gradually for a long time.

## 5 Conclusions

Long-term monitoring and follow-up of BNP to screen patients with cardiac insufficiency could be practical. During or after radiotherapy, myocardial fibers were damaged, which indirectly reflected the sensitivity of cardiomyocytes to radiation. Consequently, BNP could be a biomarker of cardiac damage at high heart absorbed doses according to radiotherapy, especially for left breast cancer patients.

## Author contributions

**Data curation:** Dan Shi.

**Formal analysis:** Cheng Zhang, Dan Shi.

**Investigation:** Dan Shi.

**Methodology:** Cheng Zhang, Dan Shi.

**Writing – original draft:** Cheng Zhang, Dan Shi.

**Writing – review & editing:** Cheng Zhang, Dan Shi, Ping Yang.
